# Unmutated but T cell dependent IgM antibodies targeting *Streptococcus suis* play an essential role in bacterial clearance

**DOI:** 10.1371/journal.ppat.1011957

**Published:** 2024-01-19

**Authors:** Dominic Dolbec, Mélanie Lehoux, Alexis Asselin de Beauville, Astrid Zahn, Javier Marcelo Di Noia, Mariela Segura

**Affiliations:** 1 Research Group on Infectious Diseases in Production Animals (GREMIP) and Swine and Poultry Infectious Diseases Research Center (CRIPA), Department of Pathology and Microbiology, Faculty of Veterinary Medicine, University of Montreal, Saint-Hyacinthe, Quebec, Canada; 2 Institut de Recherches Cliniques de Montréal, Center for Immunity, Inflammation and Infectious Diseases, Quebec, Canada; 3 Department of Medicine, Faculty of Sciences, University of Montreal, Montreal, Quebec, Canada; University of Washington, UNITED STATES

## Abstract

*Streptococcus suis* serotype 2 is an important encapsulated bacterial swine pathogen and zoonotic agent for which no effective vaccine exists. The interaction with B cells and the humoral response against *S*. *suis* are poorly understood despite their likely relevance for a potential vaccine. We evaluated germinal center (GC) B cell kinetics, as well as the production and role of *S*. *suis*-specific antibodies following infections in a mouse model. We found that mice infected with *S*. *suis* developed GC that peaked 13–21 days post-infection. GC further increased and persisted upon periodic reinfection that mimics real life conditions in swine farms. Anti-*S*. *suis* IgM and several IgG subclasses were produced, but antibodies against the *S*. *suis* capsular polysaccharide (CPS) were largely IgM. Interestingly, depletion of total IgG from the wild-type mice sera had no effect on bacterial killing by opsonophagocytosis *in vitro*. Somatic hypermutation and isotype switching were dispensable for controlling the infection or anti-CPS IgM production. However, T cell-deficient (*Tcrb-/-*) mice were unable to control bacteremia, produce optimal anti-CPS IgM titers, or elicit antibodies with opsonophagocytic activity. SAP deficiency, which prevents GC formation but not extrafollicular B cell responses, ablated anti *S*. *suis*-IgG production but maintained IgM production and eliminated the infection. In contrast, B cell deficient mice were unable to control bacteremia. Collectively, our results indicate that the antibody response plays a large role in immunity against *S*. *suis*, with GC-independent but T cell-dependent germline IgM being the major effective antibody specificities. Our results further highlight the importance IgM, and potentially anti-CPS antibodies, in clearing *S*. *suis* infections and provide insight for future development of *S*. *suis* vaccines.

## Author summary

*Streptococcus suis* is an important bacterial pathogen that affects swine and can cause severe infections in humans. To date, there is no efficacious vaccine commercially available to prevent *S*. *suis* infections. To make it worse, little is known on how the bacterium interacts with B cells, which are the cells that produce protective antibodies. In this work, we sought to better understand how antibodies are produced following infection, what is their specific targets and which antibodies play an important role in the elimination of the bacteria. To do so, we first performed infection and immunisation trials in mice to characterize how the antibody response develops. Following this, we tested mice of various genetic profiles to evaluate the role of important genes and cell types in the production of useful antibodies. The results obtained in this study indicate that IgM antibodies, such as the ones able to target the protective shell of the bacteria, play an important role in *S*. *suis* elimination. The acquired knowledge in this study provides a better understanding of how the immune response develops against *S*. *suis* and brings insight for future development of vaccines against *S*. *suis*.

## Introduction

*Streptococcus suis* is one of the most important bacterial pathogens in swine production worldwide [1], responsible of heavy economic losses for the industry and causing severe health conditions such as septicemia, meningitis, and death of affected pigs [2]. *S*. *suis* is also a zoonotic agent that can represent a health risk for the workers of the swine industry [3]. Human infection usually leads to the development of septicemia, meningitis, and other severe systemic diseases. Deadly outbreaks in Asia caused by particularly virulent strains have been reported in the past [1].

*S*. *suis* is a Gram-positive bacterium, that mainly colonizes the upper respiratory tract of swine, particularly the tonsils [4]. *S*. *suis* classification into 29 distinct serotypes is based on the antigenic diversity of the thick capsular polysaccharide (CPS) that surrounds the bacterium [5]. Of all the serotypes described, the serotype 2 is the most clinically important in both pigs and humans [1]. Transmission of bacteria is done vertically when piglets get in contact with the contaminated vaginal secretions from the sow and, horizontally in the herd via aerosols and direct contact [4]. Transmission of new virulent strains to the farm can occur via the introduction of healthy carriers to the herd [6]. Horizontal transfer is exacerbated during outbreaks where diseased animals shed higher amounts of bacteria [2]. In humans, the main reported route of entry is via small skin wounds and, in some Asian countries, via consumption of raw or undercooked contaminated pork products [3].

Prevention and treatment of *S*. *suis* infections heavily relies on the use of antimicrobials. The rise of antimicrobial resistances in *S*. *suis* is alarming [7] and this bacterium represents a high risk of transferring resistances to other animal and human pathogens [8]. Vaccination is a powerful tool to prevent infections and reduce antimicrobial use. However, no efficient universal vaccine is commercially available to prevent *S*. *suis* infections despite of several studies and trials exploring many potential candidate antigens [9–11]. Multiple factors are responsible for this, such as the high genomic diversity between strains and the presence of multiple serotypes. The lack of fundamental knowledge on the adaptive immune response induced by *S*. *suis* infection adds to the challenge of developing an efficacious vaccine.

Immune evasion capabilities have been attributed to the virulence factors of *S*. *suis* [12]. From this arsenal, the thick CPS is widely recognized as the most important virulence factor that allows bacterial evasion of the host immune system and favors bloodstream dissemination by interfering with *S*. *suis* phagocytosis and killing by host phagocytes [12,13]. Indeed, it has been demonstrated that the CPS of *S*. *suis* serotype 2 hinders phagocytosis and cytokine release mediated by dendritic cells (DC) [14] and affects optimal T cell activation [15] allowing the bacteria to interfere with proper antigen presentation and downstream primary and memory responses of T cells. Studies on *S*. *suis*, other streptococci and encapsulated bacteria have found anti-CPS antibodies to be important actors of immunity that enable opsonisation and elimination of pathogenic bacteria [16–20], making the generation of these antibodies highly desirable when designing vaccines.

Antibodies can be produced via T-dependent (TD) responses, usually when follicular (FO) B cells recognize cognate antigens and receive help from cognate CD4^+^ T cells [21] Antigen-activated B cells can then express the enzyme Activation induced deaminase (AID), which initiates a genetic modification of the antibody genes to allow isotype change, for instance from IgM to IgG, by the process of class switch recombination (CSR) [22–24]. Regardless of switching, a proportion of the activated B cells in TD responses can form germinal centers (GCs), where AID activity will enable affinity maturation of the antibody response by initiating the process of somatic hypermutation (SHM) [24,25]. GCs are specialized microenvironments formed upon infection inside secondary lymphoid organs. GC are organized into functionally distinct dark zone (DZ) and a light zone (LZ) [21,25]. B cells in the DZ undergo SHM, which will change the affinity for their cognate antigen by introducing mutations in the exon encoding the variable region of the antibody. In the LZ, the B cells will be selected by interactions with T cells, according to the affinity of their antibody molecules. The GC can thus increase the overall affinity of the antibody response [21,25]. High affinity and class-switched GC B cells tend to generate plasma cells (PC), while memory B cells present a range of affinities and isotypes. Together, high-affinity PC and memory B cells ensure a long-lasting antibody response [21,25].

On the other hand, immune responses against polysaccharides, such as the CPS, do not usually depend on the help of cognate T cells and can be triggered in a T cell-independent (TI) fashion [26]. Marginal zone (MZ) B cells live in a form of pre-activated state and are the main responders to TI antigens [27]. The binding of polysaccharides to the B cell receptor (BCR) can result in a strong signal, due to the highly repetitive structure of polysaccharides, that can induce BCR cross-linking [28], which will trigger rapid differentiation and proliferation of reactive MZ B cells into antibody-secreting cells [29]. However, research on the immunogenicity of CPS antigens from *S*. *suis* has found them to be mostly poorly immunogenic [30–32].

As aforementioned, to this day, there is still no efficacious vaccine commercially available to prevent *S*. *suis* infections. Surprisingly little is known on the interactions between *S*. *suis* and B cells and the subsequent development of the adaptive humoral response. To address this need, our objective was to properly characterize the adaptive immune response following *S*. *suis* infection by monitoring GC formation, antibody production and maturation, as well as antibody functionality to identify the source of effective antibodies contributing to eliminating *S*. *suis*, which will inform vaccine development strategies.

## Results

### *Streptococcus suis* infection induces robust antibody production

**Fig 1:**
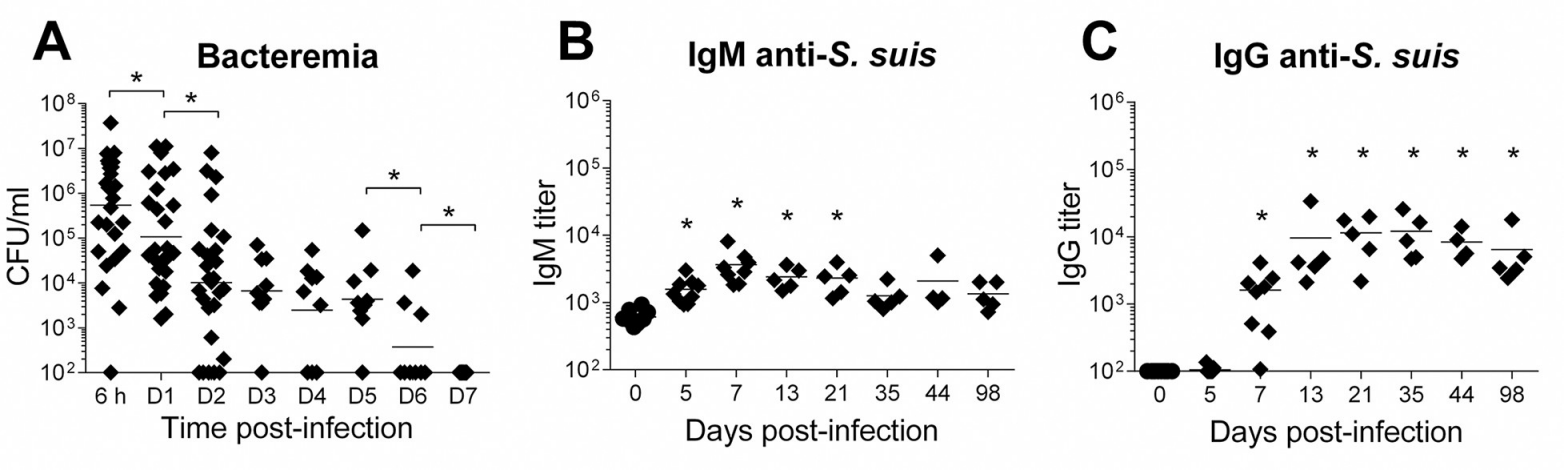
*Streptococcus suis* primary infection induces a persistent antibody response. C57BL/6 WT mice were infected with a 1 x 10^6^ CFU dose of live *S*. *suis* serotype 2 strain P1/7. (A) Bacterial blood burden of mice at various times post-infection (*n*; 6 h = 28, D1 = 27, D2 = 27, D3 = 9, D4 = 9, D5 = 9, D6 = 9 and D7 = 9). Data are represented as individual mouse values with geometric means. (B) Anti-*S*. *suis* IgM and (C) IgG titers in the serum of mice, measured at various time points by ELISA (*n*; D0 = 10, D5 = 9, D7 = 8, D13 = 5, D21 = 5, D35 = 5, D44 = 4 and D98 = 5). Data are represented as individual values with mean. * *p* < 0.05, indicates a significant difference between indicated time points (A) or with time 0 (B), as evaluated by Student’s *t*-test.

To characterize how B cells respond to an infection by *S*. *suis*, C57BL/6 wild-type (WT) mice were infected with live *S*. *suis* serotype 2. Following infection, bacterial blood burden was analyzed at different time points to evaluate the host ability to eliminate the bacteria during a single primary infection. Bacterial levels in blood significantly declined by several logs in the first 48 h **(Fig 1A)**; yet substantial bacteremia persisted at 48 h, which correlates with the onset of clinical signs [33]. Bacteremia remained steady for 5 days and became largely undetectable only by day 7 (**Fig 1A)**, in contrast to a non-encapsulated strain, which is cleared within 6 h post-infection [34].

The production of anti-*S*. *suis* specific antibodies was evaluated using whole bacteria as coating antigen. As shown in **Fig 1B**, specific anti-*S*. *suis* IgM and IgG antibodies are produced and are still detected 98 days after the primary infection. IgM production peaked early by day 5 and was significantly increased until day 21 post-infection before slowly decreasing to near-basal levels by day 35. Meanwhile, IgG production was significantly increased on day 7 but reached maximal levels at day 13 and persisted at similar levels at least until day 98.

These results show that a single infection with *S*. *suis* can induce the production of bacterium-specific IgM and IgG antibodies, but their kinetics of production, notably that of IgG, trail after infection clearance.

### *Streptococcus suis* primary infection stimulates the formation of germinal centers

**Fig 2:**
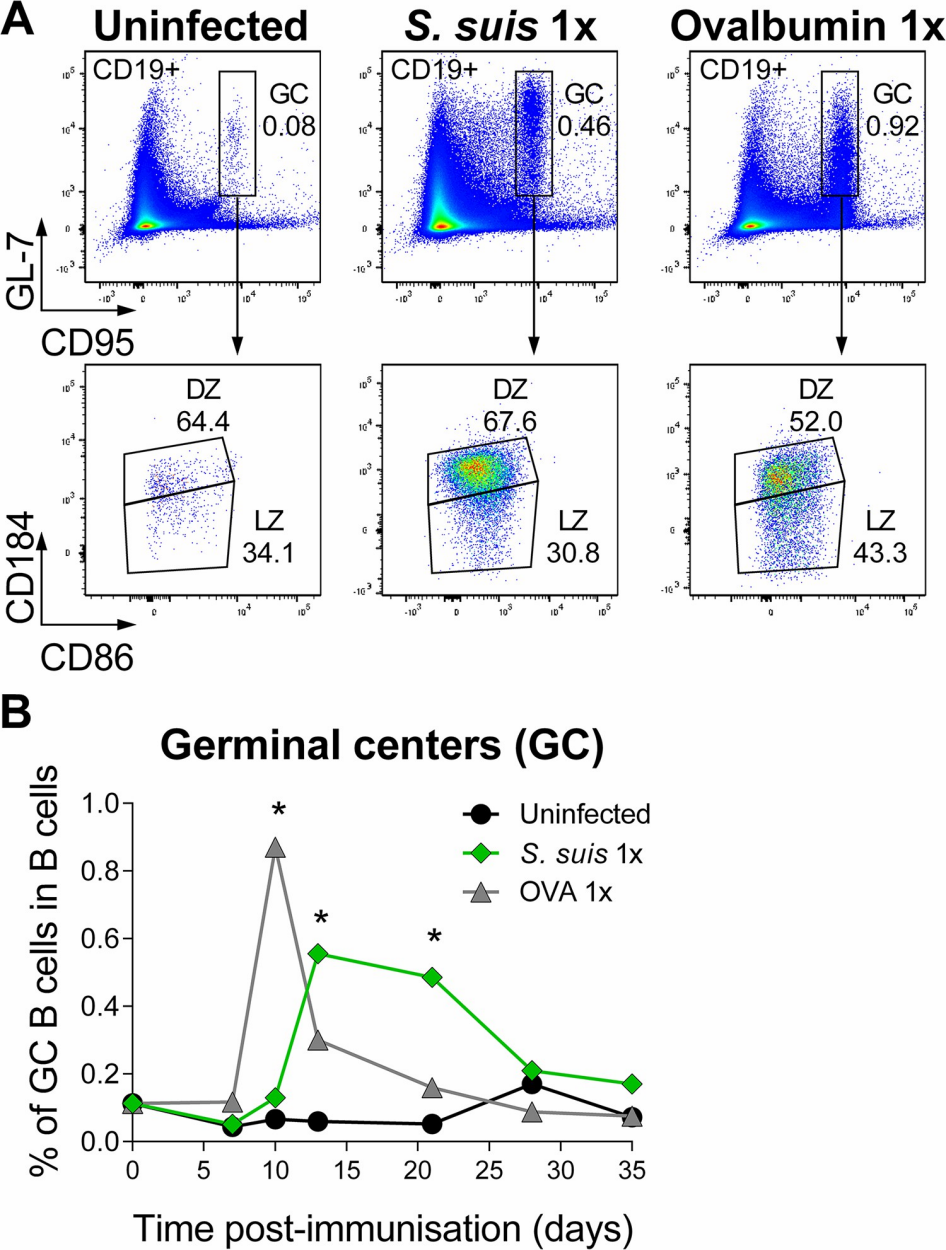
*Streptococcus suis* infection generates germinal centers. C57BL/6 WT mice were infected with a 1 x 10^6^ CFU dose of live *S*. *suis* serotype 2 strain P1/7. (A) Representative flow cytometry plots of germinal center (GC) B cells (gated on CD19+ cells) and corresponding dark zone (DZ) and light zone (LZ) GC B cells in the spleens of mice uninfected, or 13 days post *S*. *suis*-infection, or 10 days after ovalbumin (OVA) one dose-immunization (1x) (for detailed information on gating, refer to S1 FIG). (B) Proportion of GC B cells evaluated by flow cytometry in the splenic B cell population of mice treated as in (A) at various time points. Data are represented as the median value from mice uninfected (*n*; D0 = 8, D7 = 6, D10 = 6, D13 = 3, D21 = 6, D28 = 3 and D35 = 4), *S*. *suis*-infected (*n*; D0 = 8, D7 = 5, D10 = 5, D13 = 18, D21 = 16, D28 = 5 and D35 = 5) and OVA-immunized (*n*; D0 = 8, D7 = 8, D10 = 10, D13 = 3, D21 = 7, D28 = 5 and D35 = 3). * *p* < 0.05, indicated a significant difference with uninfected mice at the corresponding time, as evaluated by Student’s *t*-test.

To further characterise the antibody response to a primary *S*. *suis* infection we determined GC formation and their kinetics in the spleens of infected animals. Uninfected and mice immunized with the model protein antigen ovalbumin (OVA), served as controls. The proportion of total splenic B cells (CD19+) was similar and remained relatively stable over time for all mouse groups **(S2A Fig)**. The *S*. *suis*-infected mice generated GC B cells (CD19^+^, GL7^+^, CD95^+^; gating strategy in **S1 Fig**), peaking at day 13 and persisting until day 21 post-infection **(Figs 2B and S2B)**. This GC peak was broader than the GC reaction against OVA, which showed a narrow peak at day 10 **(Figs 2B and S2B)**. The GC induced by *S*. *suis* infection showed a similar DZ (CD19^+^, Gl7^+^, CD95^+^, CD184^+^ and CD86^int^) to LZ (CD19^+^, Gl7^+^, CD95^+^, CD184^-^ and CD86^+^) ratio than low levels of spontaneous GC present in naive mice but was different from GC induced by OVA (**Fig 2A**).

The results obtained demonstrate that the initial *S*. *suis* infection induces the formation of GC in the spleen, with a delayed but more persistent kinetics than immunization with a protein. It is also important to notice that the GC reaction peaks after the bacteremia is resolved.

### Antibodies targeting *Streptococcus suis* help to control re-infections

**Fig 3:**
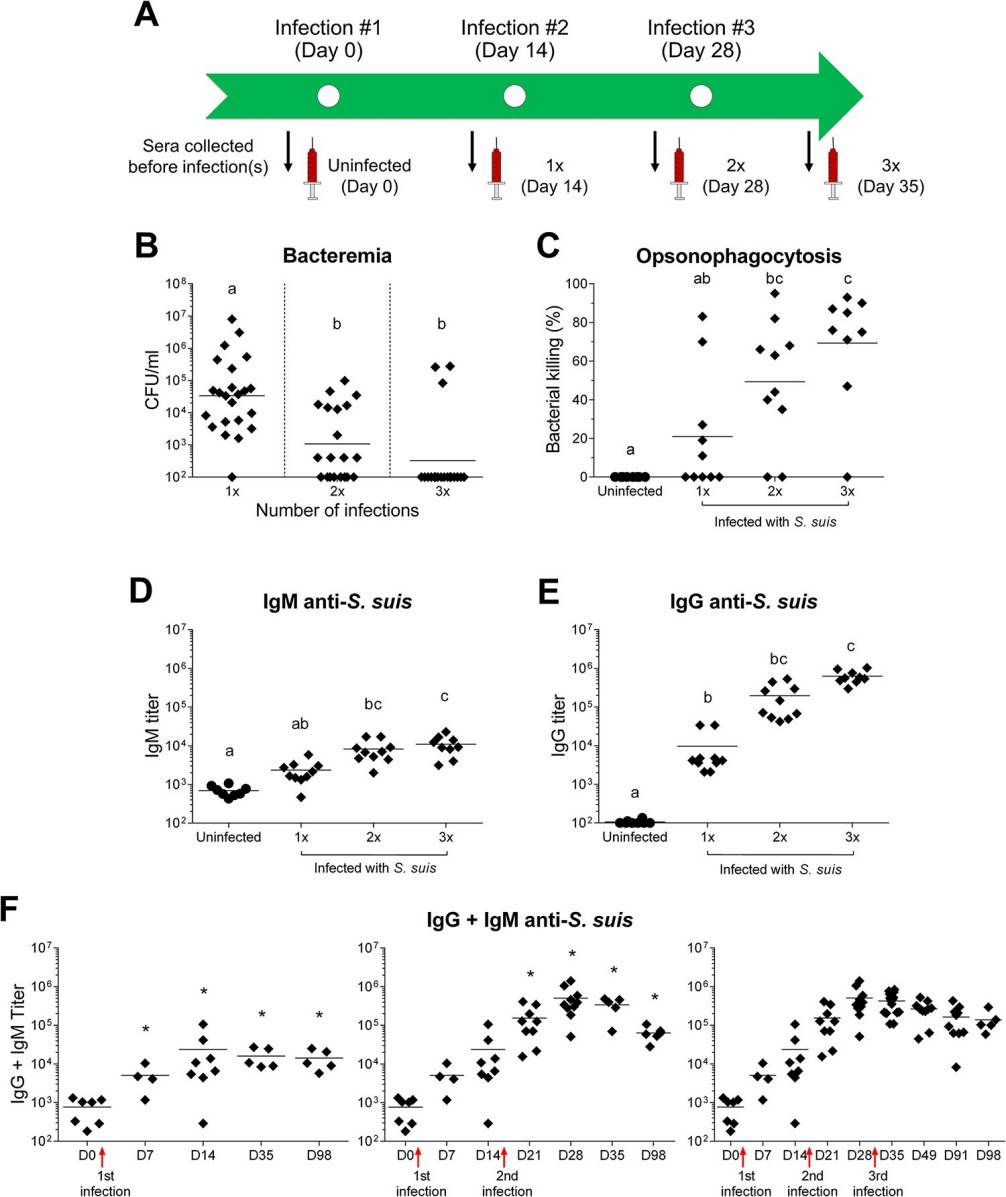
Repeated *Streptococcus suis* infections induce antibodies with opsonophagocytic activity. C57BL/6 WT mice were infected with successive 1 x 10^6^ CFU doses of live *S*. *suis* serotype 2 strain P1/7. (A) Timeline of mouse infections and serum collection for opsonophagocytosis killing assay (OPA). (B) Bacterial blood burden in mice 24 h after the first, second and third (*n;* 1x = 23, 2x = 20 and 3x = 19) *S*. *suis* infection. Data are represented as individual values with geometric means. (C) Mouse serum-induced opsonophagocytosis killing of *S*. *suis*, evaluated by OPA. Mouse sera (*n*; uninfected = 8, 1x = 10, 2x = 10 and 3x = 9) were collected according to the timeline presented in (A) and data are represented as individual values with mean. Anti-*S*. *suis* (D) IgM titers and (E) anti-*S*. *suis* IgG titers of the mouse serum samples tested in the OPA. Data are represented as individual values with mean. (F) Anti-*S*. *suis* IgG + IgM antibody titers in the sera of mice infected 1x (*n;* D0 = 7, D7 = 4, D14 = 8, D35 = 5 and D98 = 5), 2x (*n;* D0 = 7, D7 = 4, D14 = 8, D21 = 9, D28 = 10, D35 = 5 and D98 = 6) and 3x (*n;* D0 = 7, D7 = 4, D14 = 8, D21 = 9, D28 = 10, D35 = 17, D49 = 10, D91 = 10 and D98 = 5) tested at various time points by ELISA. (B-F) Data are represented as individual values with mean. (B-E) Groups not sharing letters (a, b or c) are significantly different from each other (*p* < 0.05) as evaluated by one-way ANOVA. (F) * *p* < 0.05 indicates a significant difference with the first time point, prior to the latest infection, as evaluated by Student’s *t*-test.

Swine are repeatedly exposed to *S*. *suis* on the farm since the bacterium is easily transmitted between animals via direct contact and aerosols. Additionally, *S*. *suis* is a normal inhabitant of the upper respiratory tract, where it can persist for long periods of time [6]. Knowing that pathogen-specific antibodies were produced following a primary *S*. *suis* infection, we evaluated the role of these antibodies in the advent of re-infections. C57BL/6 WT mice were sequentially infected with equal doses of live *S*. *suis* at 14 day-intervals and bled 24 h after each infection **(Fig 3A)**. Bacterial blood burden was reduced to a significantly larger extent with each re-infection **(Fig 3B)**, suggesting an evolving adaptive response.

To determine if antibodies might contribute to the elimination of *S*. *suis*, sera samples were collected as indicated in **Fig 3A** and tested in an *in vitro* opsonophagocytosis assay (OPA). Sera collected from mice infected 2x or 3x were significantly more efficient to induce the *in vitro* opsonophagocytic killing of *S*. *suis* than sera after primary infection, showing that opsonophagocytic activity increased with each infection **(Fig 3C)**.

The serum samples tested in OPA were also evaluated for anti-*S*. *suis* specific IgM and IgG antibody content. Correlating with the OPA results, IgM and IgG serum antibody levels continued to increase after each infection **(Fig 3D and 3E).** To further characterize antibody production following successive infections, serum samples were analyzed at various time points after each (re-)infection **(Fig 3F)**. The second infection significantly increased the production of antibodies targeting *S*. *suis* compared to the initial infection. However, a third infection was not able to further increase the antibody titer.

Taken together, these results suggest that anti-*S*. *suis* antibodies that can induce bacterial killing are generated upon infection and reach maximal levels after one re-infection in this model, which likely helps controlling the infection.

### Titers of all anti-*S suis* IgG subclasses increase without affinity maturation during re-infections

**Fig 4:**
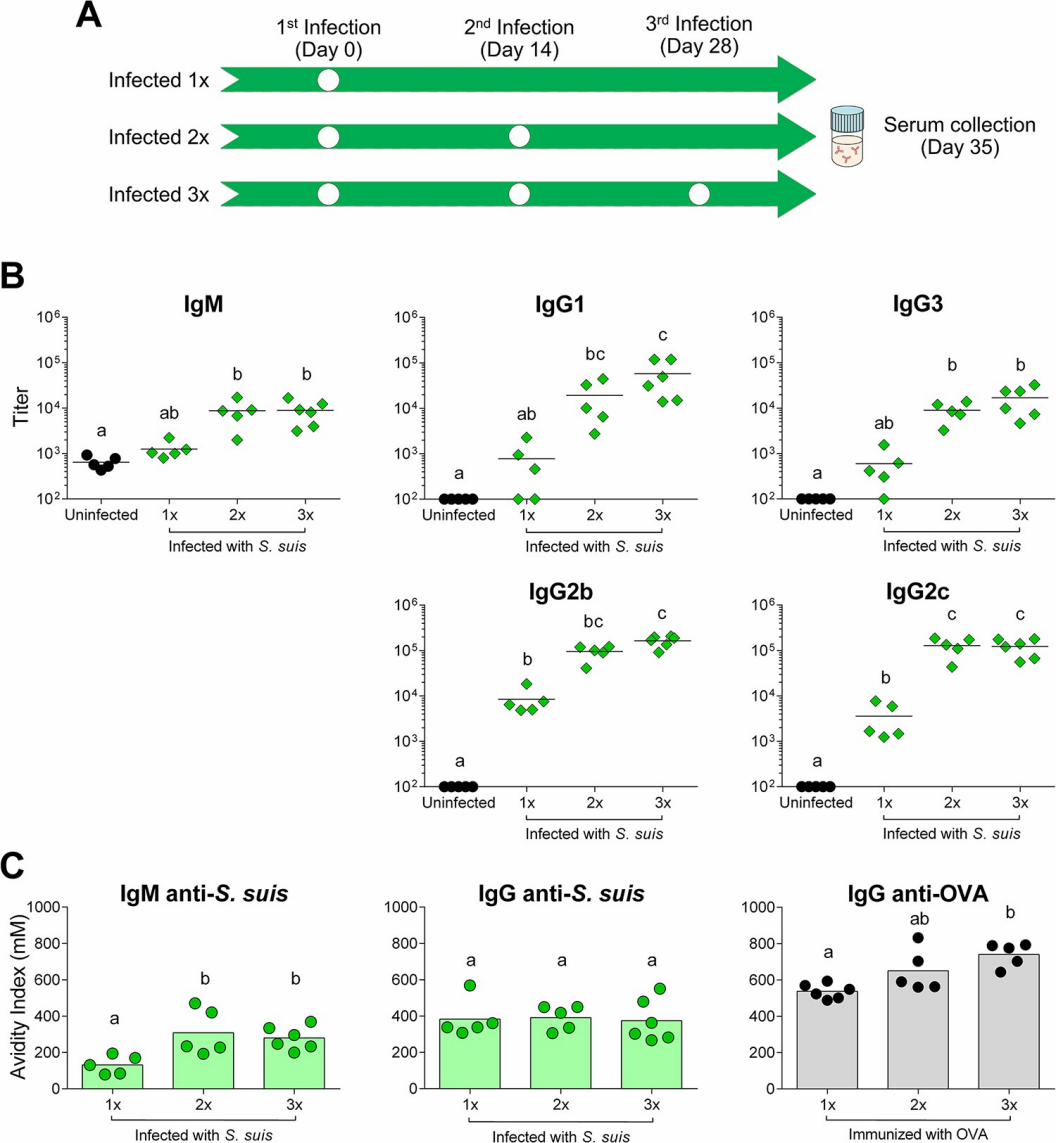
Anti-*Streptococcus suis* IgG titers, but not their avidity, increase with repeated infections. Groups of C57BL/6 mice were infected with 1 x 10^6^ CFU doses of live *S*. *suis* serotype 2 strain P1/7. (A) Timeline of the infections performed in the different groups of mice with serum collection on day 35 for all groups. (B) Anti-*S*. *suis* titers of IgM, IgG1, IgG2b, IgG2c and IgG3 in the sera of uninfected mice (*n* = 5) or mice infected 1x (*n* = 5), 2x (*n* = 5) or 3x (*n* = 6), measured by ELISA. Data are represented as individual values with mean. (C) Avidity index of *S*. *suis*-specific IgMs and IgGs in the sera of mice infected 1x (*n* = 5), 2x (*n* = 5) or 3x (*n* = 6), and of ovalbumin (OVA)-specific IgGs in the sera of mice immunized 1x (*n* = 6), 2x (*n* = 5) or 3x (*n* = 5), evaluated by ELISA coupled to the use of the chaotropic agent NaSCN. The avidity index represents the mM concentration of NaSCN at which 50% of antigen-specific antibodies are displaced. Data are represented as individual values with mean. Groups not sharing letters (a, b or c) are significantly different from each other (*p* < 0.05) as evaluated by one-way ANOVA.

Having determined that anti-*S*. *suis* antibody production and functionality increased with re-infections, we decided to further dissect the quality of the antibody response. Groups of C57BL/6 WT mice were infected 1x, 2x, or 3x, and sera collected on day 35 for all groups **(Fig 4A)**. The primary *S*. *suis* infection induced significant levels of bacterium-specific IgG2b and IgG2c, but relatively lower levels of IgM, IgG1 and IgG3. Upon re-infection (2x mice) IgM and IgG3 targeting the bacteria modestly but significantly increased, while IgG1, IgG2b and IgG2c increased more substantially **(Fig 4B)**. Another reinfection (3x mice) did not significantly increase any isotype **(Fig 4B)**, indicating that a single re-infection was sufficient to saturate the response.

We then asked if the increase in anti-*S*. *suis* antibodies correlated with an increase in their avidity. The avidity of anti-*S*. *suis* IgM significantly increased upon one re-infection when compared to IgM generated against the primary infection but did not further increase in mice infected 3x (**Fig 4C**). In contrast, the anti-bacterial IgG response did not show any significant avidity improvement, regardless of the number of infections (**Fig 4C**). Similar results were observed when individually analyzing the IgG subclasses **(S3 Fig)**. As expected, the anti-OVA IgG showed an increase in avidity after boosting **(Fig 4C)**.

The results showed that one reinfection with *S*. *suis* is sufficient to increase the titers of anti-bacterium IgM and IgGs to their maximal levels, but while the affinity of IgM undergoes some apparent maturation, for the IgG it does not.

### *Streptococcus suis* reinfections sustain a persistent germinal center reaction

**Fig 5:**
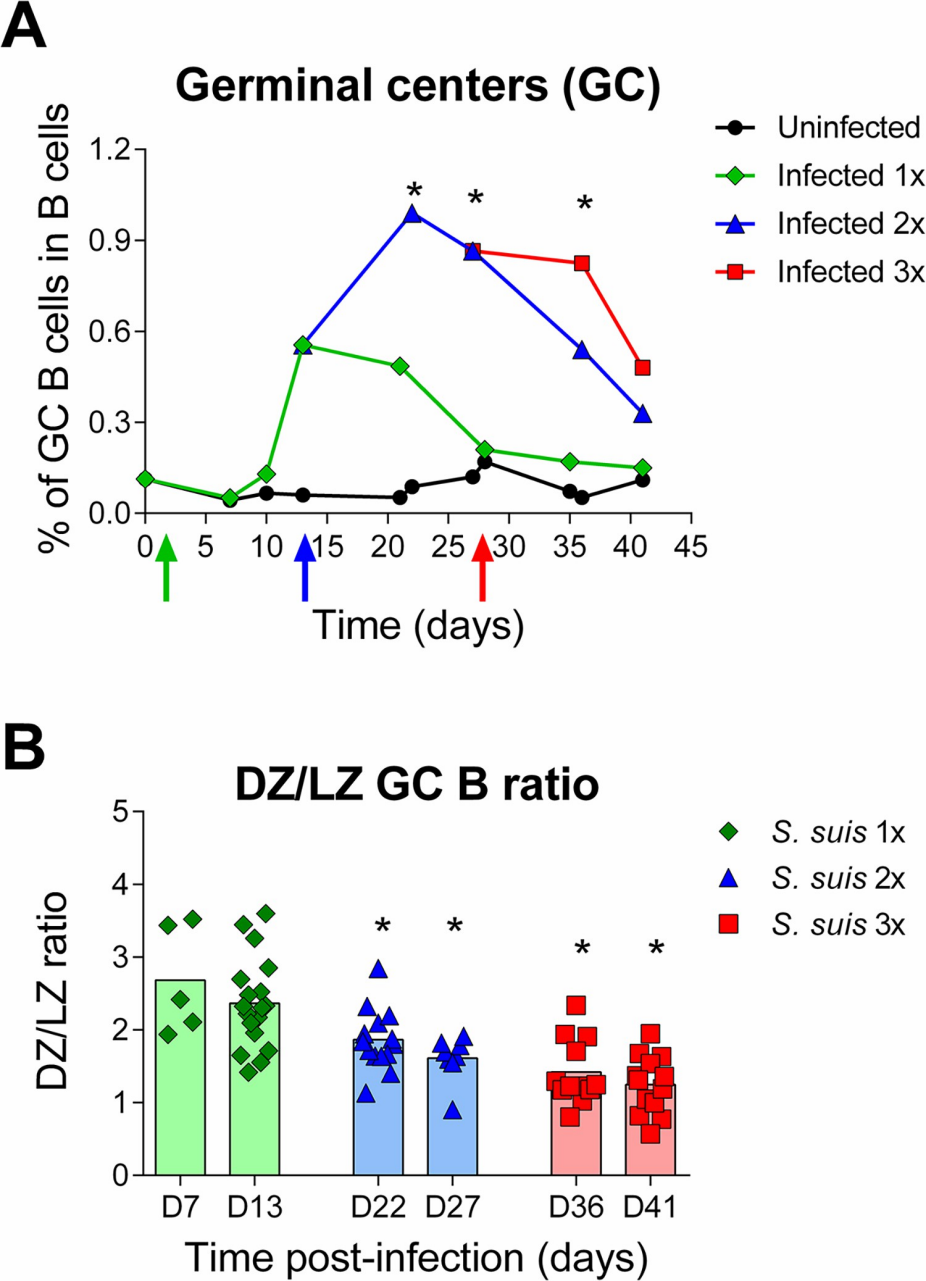
*Streptococcus suis* reinfections sustain the germinal center reaction. C57BL/6 mice were infected with 1 x 10^6^ CFU doses of live *S*. *suis* serotype 2 strain P1/7. (A) Proportion of GC B cells in the splenic B cell population evaluated by flow cytometry at various time points in uninfected mice (*n*; D0 = 8, D7 = 6, D10 = 6, D13 = 3, D21 = 6, D22 = 5, D27 = 5, D28 = 3, D35 = 4, D36 = 3 and D41 = 3), mice infected with *S*. *suis* 1x (*n*; D0 = 8, D7 = 5, D10 = 5, D13 = 18, D21 = 16, D28 = 5, D35 = 5 and D41 = 6), 2x (*n*; D22 = 14, D27 = 8, D36 = 5 and D41 = 5) and 3x (*n*; D36 = 12 and D41 = 13). Data are represented as median. (B) GC B cell Dark zone (DZ) vs Light zone (LZ) ratios of mice infected with *S*. *suis* once (*n*; D7 = 5 and D13 = 18), twice (*n*; D22 = 14 and D27 = 8) and thrice (*n*; D36 = 12 and D41 = 13). Data were analyzed by Student’s *t*-test where * indicates significant differences (*p* < 0.05) with the group infected 1x.

We then asked what was the effect of re-infection on the generation of GC B cells. C57BL/6 WT mice were infected 1x, 2x, or 3x and splenic GC B cells determined by flow cytometry. Mice reinfected 2x produced ~2-fold more GC B cells than the peak GC of mice with a single infection (*p* < 0.05) **(Fig 5A)**. Another re-infection did not further increase GCs, but it sustained the reaction **(Fig 5A)**. In each case the GC reaction started decreasing ~8–10 days after the last infection dose **(Fig 5A)**, suggesting that GC resolve once the bacteria are cleared from the blood. The DZ to LZ ratio of GC B cells was significantly (*p* < 0.05) reduced in re-infected mice compared with mice responding to a primary infection **(Fig 5B)**.

These results indicate that regular reinfections maintain a strong and sustained GC reaction that evolves over time.

### SHM and CSR are dispensable to produce opsonophagocytic antibodies to *Streptococcus suis*

The production of anti-*S*. *suis* antibodies of multiple isotypes, and the apparent increase in anti-*S*. *suis* IgM affinity would implicate CSR and SHM in the response. We asked if isotype switching and/or affinity maturation contributed to the generation of effector antibodies by comparing the response of *Aicda*^-/-^ mice, which are unable to do either SHM or CSR [23], and *Ung*^-/-^ mice, which have reduced CSR, albeit this can be compensated by chronic antigen exposure, but can undergo SHM and affinity maturation of IgM [35,36].

**Fig 6:**
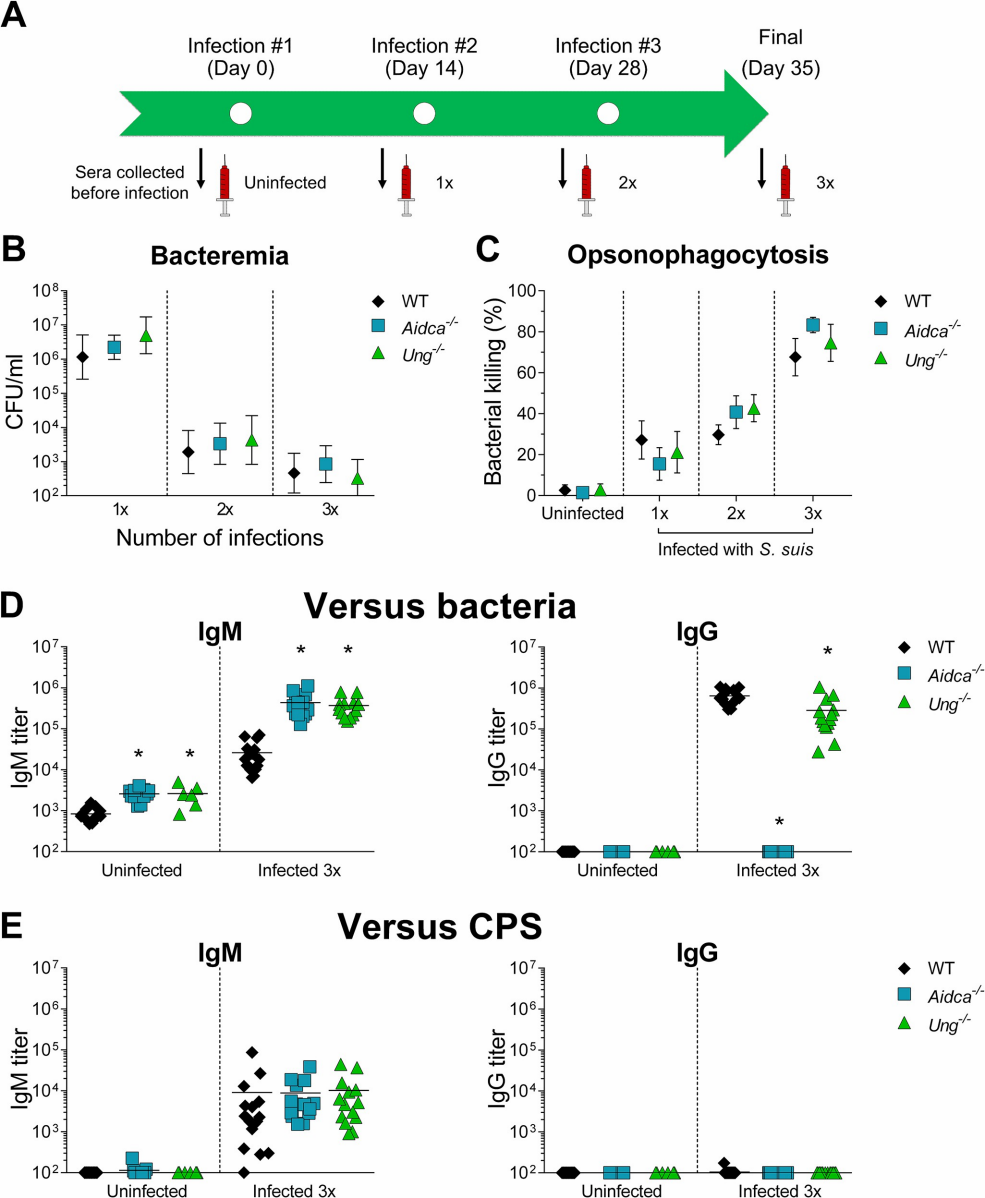
AID and UNG are dispensable for the production of opsonophagocytic anti-*Streptococcus suis* antibody. C57BL/6 WT, *Aidca*^*-/-*^ and *Ung*^*-/-*^ mice were infected with 1 x 10^6^ CFU doses of live *S*. *suis* serotype 2 strain P1/7. (A) Timeline of the infections performed in the different groups of mice with collection times of sera used in opsonophagocytosis killing assay (OPA). (B) Bacterial blood burden of mice 24 h after the first (*n*; WT = 27, *Aidca*^*-/-*^ = 33 and *Ung*^*-/-*^ = 22), second (*n*; WT = 17, *Aidca*^*-/-*^ = 18 and *Ung*^*-/-*^ = 14) and third (*n*; WT = 17, *Aidca*^*-/-*^ = 14 and *Ung*^*-/-*^ = 14) *S*. *suis* infection. Data are represented as geometric means and 95% confidence interval. (C) Mouse serum-induced opsonophagocytosis killing of *S*. *suis*, evaluated by OPA for WT, *Aidca*^*-/-*^ and *Ung*^*-/-*^ sera (*n*; uninfected = 5, infected 1x = 5, infected 2x = 5 and infected 3x = 5). Tested sera were collected on the timepoints presented in (A). Data are represented as mean ± SEM. (D) Anti-*S*. *suis* IgM and IgG titers in sera collected on day 35 from uninfected mice (*n*; WT = 13, *Aidca*^*-/-*^ = 11 and *Ung*^*-/-*^ = 6) or mice infected three times (*n*; WT = 16, *Aidca*^*-/-*^ = 14 and *Ung*^*-/-*^ = 14), measured by ELISA. Data are represented as individual values with mean. (E) Anti-*S*. *suis* capsular polysaccharide (CPS) IgM and IgG titers in sera collected on day 35 from uninfected mice (*n*; WT = 13, *Aidca*^*-/-*^ = 11 and *Ung*^*-/-*^ = 6) or mice infected three times (*n*; WT = 17, *Aidca*^*-/-*^ = 14 and *Ung*^*-/-*^ = 14), measured by ELISA. Data are represented as individual values with mean. Data were analyzed by Student’s *t*-test where * indicates significant differences with WT mice (*p* < 0.05).

Mice were infected 1x, 2x, or 3x and serum collected at different time post-infection(s) and on day 35 for all groups **(Fig 6A)**. No significant differences in the bacteremia were observed between WT and AID- or UNG (uracil DNA glycosylase)-deficient mice **(Fig 6B)**. To complement our *in vivo* observations, sera samples were collected and tested *in vitro* by the OPA test. Again, no significant differences in bacterial killing capacity were observed between the sera of WT and either mutant mouse **(Fig 6C)**. As expected, anti-*S*. *suis* response was exclusively IgM in *Aidca*^-/-^ mice, while *Ung*^-/-^ mice showed a similarly enhanced anti-*S*. *suis* IgM response and significantly reduced IgG (2-fold lower than WT mice) **(Fig 6D)**. To complete the characterization of the antibody response, we measured the levels of antibodies specifically targeting the CPS of *S*. *suis* **(Fig 6E)**. All mouse lines produced exclusively anti-CPS IgM at the same levels **(Fig 6E)**.

These results demonstrate that SHM or CSR are not required to produce the antibodies able to opsonize and induce phagocytosis of *S*. *suis* and that are most likely to contribute to the elimination bacteria upon reinfection.

### IgM antibodies play an essential role in *Streptococcus suis* elimination

To critically test the hypothesis that antibodies played a role in bacterial elimination, we resorted to mice that allowed us to dissect the contribution of T cells, B cells and GC in the anti-*S*. *suis* response. We used *Tcrb*^*-/-*^ mice, lacking α/β T cells [37], μMT mice, lacking all B cells [38], and *Sh2d1a*^*-/Y*^ mice, who are deficient for SAP (signaling lymphocyte activation molecule or SLAM-associated protein) and fail to produce GC but can still mount extrafollicular B cell responses [39].

**Fig 7:**
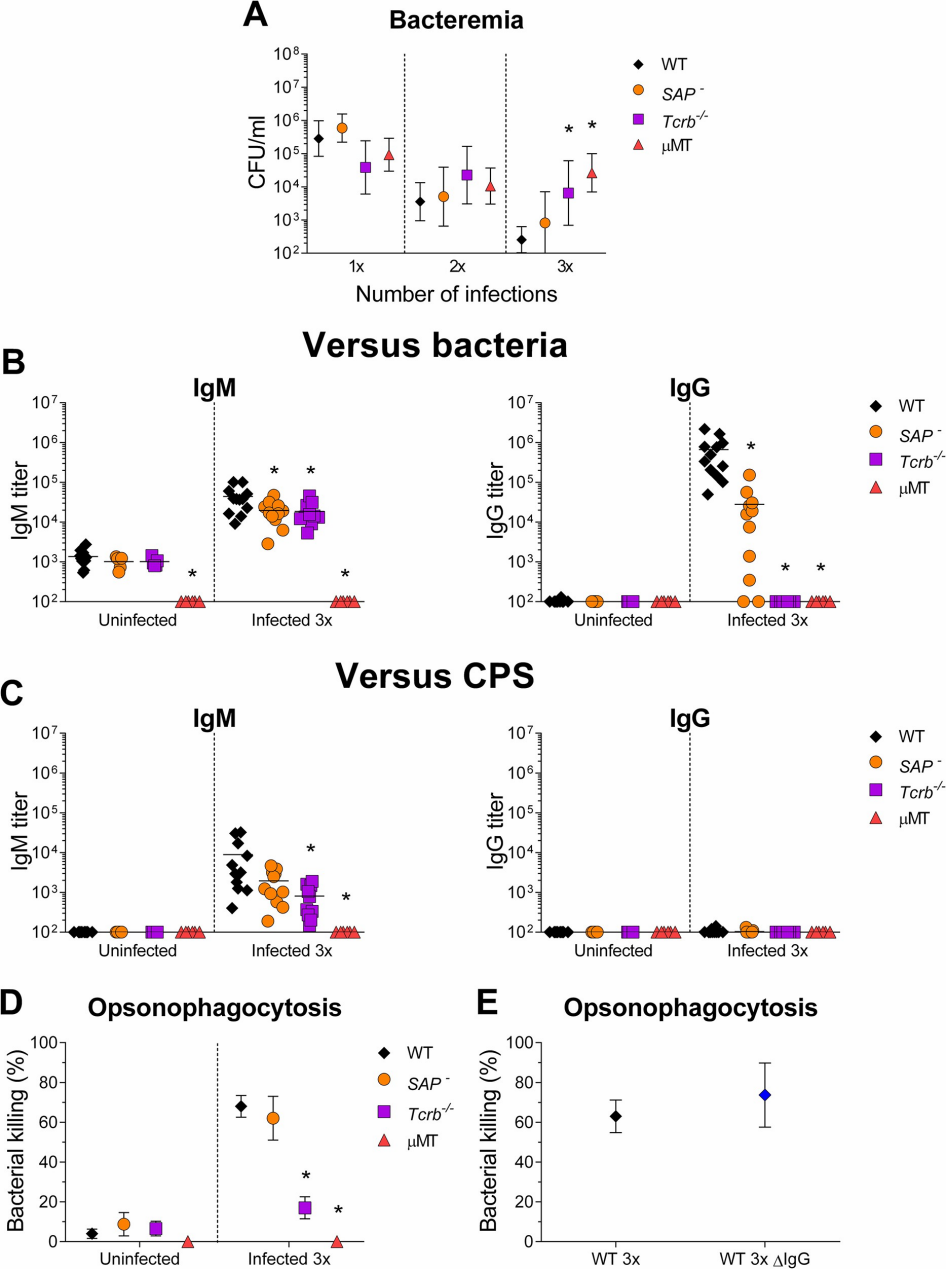
Germinal center independent anti-capsular IgMs play an essential role in the elimination of *Streptococcus suis*. WT, SAP^-^ (*Sh2d1a*^*-/Y*^), *Tcrb*^*-/-*^ and μMT mice of C57BL/6J lineage were infected with 1 x 10^6^ CFU doses of live *S*. *suis* serotype 2 strain P1/7. (A) Bacterial blood burden of mice 24 h after the first (*n*; WT = 30, SAP^-^ *= 20*, *Tcrb*^*-/-*^ = 10 and μMT = 18), second (*n*; WT = 24, SAP^-^ *=* 11, *Tcrb*^*-/-*^ = 10 and μMT = 12) and third (*n*; WT = 24, SAP^-^ *= 11 Tcrb*^*-/-*^ = 10 and μMT = 12) *S*. *suis* infection. Data are represented as geometric means and 95% confidence interval. (B) Anti-*S*. *suis* titers of IgM and IgG of sera collected on day 35 from uninfected mice (*n*; WT = 10, SAP^-^ *=* 5 *Tcrb*^*-/-*^ = 5, μMT = 6) and mice infected three times (*n*; WT = 12, SAP^-^ *= 11 Tcrb*^*-/-*^ = 10, μMT = 6), measured by ELISA. Data are represented as individual values with mean. (C) Anti-*S*. *suis* capsular polysaccharide (CPS) titers of IgM and IgG in sera collected on day 35 from uninfected mice (*n*; WT = 10, SAP^-^ *= 5 Tcrb*^*-/-*^ = 5, μMT = 6) and mice infected three times (*n*; WT = 12, SAP^-^ *= 11 Tcrb*^*-/-*^ = 10, μMT = 6), measured by ELISA. Data are represented as individual values with mean. (D) Mouse serum-induced opsonophagocytosis killing of *S*. *suis*, evaluated by opsonophagocytosis killing assay (OPA) for sera collected on day 35 from uninfected mice (*n*; WT = 10, SAP^-^ *= 5 Tcrb*^*-/-*^ = 5, μMT = 6) or mice infected three times (*n*; WT = 12, SAP^-^ *= 11 Tcrb*^*-/-*^ = 10, μMT = 6). Data are represented as mean ± SEM. (E) Mouse serum-induced opsonophagocytosis killing of *S*. *suis*, evaluated by OPA for WT (*n* = 3) sera collected on day 35 from mice infected three times and WT ΔIgG sera (*n* = 3). WT ΔIgG serum was produced by depleting the IgG content from the WT sera using Dynabeads coupled with protein G. Data are represented as mean ± SEM. Data were analyzed by Student’s *t*-test where * indicates significant differences with WT mice (p < 0.05).

Mice were infected 1x, 2x, or 3x and analyzed for bacterial elimination and antibody responses **(**as displayed in **Fig 6A)**. *Tcrb*^*-/-*^ and μMT mice showed significantly impaired control of blood bacteremia when compared to WT mice but only after the third infection **(Fig 7A)**, demonstrating the need for T and B cells for controlling re-infections. On the other hand, SAP-deficient mice controlled the infection similarly to WT (**Fig 7A**), indicating that GC were not required.

*Tcrb*^*-/-*^ mice did not produce any anti-*S*. *suis* IgG, indicating that all the IgG response upon infection was T cell-dependent, **(Fig 7B)**. On the other hand, SAP-deficient mice were significantly impaired but still able to produce some anti-*S*. *suis* IgG (23-fold less than WT), revealing the existence of an extrafollicular response upon repeated reinfections **(Fig 7B).** Both *Tcrb*^*-/-*^ and SAP-deficient mice were able to produce anti-*S*. *suis* IgM, albeit at 2-fold reduced levels compared to WT **(Fig 7B**). Both mice also produced less anti-CPS IgM response, although this only reached significance in *Tcrb*^*-/-*^ mice **(Fig 7C**). As expected, a total absence of antibodies was observed in μMT mice **(Fig 7B and 7C)**.

To directly test the role of antibodies in *S*. *suis* clearance, we confirmed that sera from infected μMT mice were unable to induce bacterial killing in the OPA test. Sera from *Tcrb*^*-/-*^ mice were drastically less able to induce bacterial killing **(Fig 7D)**, indicating the relevance of T cell help in producing the opsonophagocytic antibodies. On the other hand, sera from infected SAP-deficient mice exhibited killing percentages similar to WT mice, indicating that GC were dispensable for generating opsonophagocytic antibodies (**Fig 7D**). To dissect the relative contribution of *S*. *suis*-specific IgG vs IgM, we depleted the IgG content of WT sera. Results of the OPA test showed no significant difference between the WT sera and its IgG-depleted counterpart **(Fig 7E)**.

These results showed that T-cell dependent IgM antibodies targeting the bacterium, likely with a contribution of anti-CPS IgM, play an essential role in the elimination of *S*. *suis*.

## Discussion

*Streptococcus suis* infections represent a world-wide challenge for the swine industry [1] and a health risk for both pigs and humans [3]. Preventing *S*. *suis* infections on the farm remains a problem and an economical burden for swine producers [40]. Indeed, despite multiple efforts investigating a wide array of vaccination strategies, there is still no efficacious vaccine commercially available to prevent *S*. *suis* infections [9]. Coincidently, there is still little knowledge about the interactions between B cells and *S*. *suis* and the resulting effects on antibody production.

The production of antibodies following *S*. *suis* infections in mice has been previously explored [31]; however, high lethal bacterial doses were used. The exacerbated inflammation caused during a lethal *S*. *suis* infection is known to modulate the expression of major histocompatibility complex (MHC) molecules [41] which could affect the optimal development of the adaptive immune response. Indeed, a correlation was established between severe clinical signs of *S*. *suis* disease and impaired antibody responses [15]. In the farm, pigs are continuously exposed to the bacteria, which is a natural habitant of swine upper respiratory tract [4], and this cohabitation might influence the overall response against the pathogen. To better mimic this, we adapted the mouse model of infection by using a non-lethal dose of live bacteria and shortened the time between subsequent infections. Resulting antibody production in mice followed a mixed Th1/Th2 profile similar to previous reports [31]. Despite not being the natural target of *S*. *suis*, mice have been recognized as an appropriate model to study *S*. *suis* pathogenesis since they have been found to develop similar clinical sings and/or clinical manifestations to those observed in pigs [42]. In previous publications, the characteristics of the primary *S*. *suis*-specific antibody response observed in pigs were very similar to those obtained in mice, although the pigs presented overall lower antibody titers. The swine protein-specific response was mainly composed of both IgG1 and IgG2 subclasses, indicating a clear isotype switching, as observed in mice [31]. In the present study, we further observed that antibody titers increased over time and reached maximal levels following a secondary infection in mice, but surprisingly, the avidity of produced IgGs was not increased. The measurement of IgG concentration and avidity towards a targeted antigen is often used as a correlate of protection for vaccines against pathogens, such as *Streptococcus pneumoniae* [43–45]. We hypothesized that the IgG response against *S*. *suis* was not properly matured and investigated if the formation of GCs was impaired. To our knowledge, this is the first study to evaluate GC formation following *S*. *suis* infections. GCs induced by an acute challenge usually form, expand, and then dissipate over a ~3–4 weeks period following antigen exposure [46]. The relationship between GC and blood bacteremia might also dictate the kinetics of GC formation and resolution. Although the mechanisms that lead to GC termination are not completely clear [47], it is well known that antigen is required for maintenance [47]. Since antigen (in the form of long-lived immune complexes) can be maintained by GC antigen-presenting cells for longer than the live pathogen can be detected, it is possible that GC resolution in *S*. *suis* infected-mice is triggered by the elimination of circulating bacteria, but the process takes several days after loss of detectable bacteremia because of the persistence of antigen in the GC. We also found that GCs induced following a primary *S*. *suis* infection formed much later and were less (as judged by the proportion of GC B cells) than GCs induced by the model protein antigen OVA. This could suggest that the immune system takes a relatively long time to recognize *S*. *suis* for GC formation, which could stem from its antigenic properties and/or from the presence of the CPS that impairs bacterial recognition and processing by the immune system [14,15,41], as well as from potential, yet unknown, immunosuppressive molecules in *S*. *suis*. Supressed GCs have also been observed during infections with other bacterial pathogens, such as *Ehrlichia muris* and *Salmonella enterica* [48,49]. A similar effect was also observed with *S*. *pneumoniae*, where it was discovered that intact and inactivated strains have immunosuppressive properties and could inhibit the generation of splenic GC B cells and generation of IgGs in mice co-immunized with OVA [50,51]. The inhibition of antibody production against OVA was also reported previously in mice infected with live *S*. *suis* [15]. Knowing this, it is possible that *S*. *suis* might possesses similar immunosuppressive mechanisms responsible for the delayed GCs and lack of increased avidity of produced anti-*S*. *suis* IgGs. This is surprising considering that GCs further increased with subsequent *S*. *suis* infections, as well as IgG titers, and that GC persisted with additional infections. Thus, the lack of apparent antibody affinity maturation would suggest some mechanism by which *S*. *suis* interferes with the function of the GC. It is noteworthy that *S*. *suis* can alter the secretion of cytokines by splenic CD4^+^ T cells [15], which might contribute to apparent lack of affinity maturation process. However, the underlying causes of the antibody affinity maturation defect remains to be studied. Nonetheless, our data does suggest that the IgG antibody response (which is directed against surface proteins) following *S*. *suis* is not protective, as evidence by preserved optimal OPA activity in sera having reduced or lacking IgG (*Aidca*^-/-^, *Ung*^*-/-*^, SAP-deficient, ΔIgG).

The innate immune system plays a key role in the elimination of pathogens; however, *S*. *suis* is known to evade phagocytosis by host cells [14,52,53]. This goes in line with the observed moderate reduction of blood bacterial burden in mice over the course of the first 48 h following the primary infection. The observed early IgM response, most likely extrafollicular, might help control the primary infection. A feature that deserves future studies. However, during subsequent infections, the mice had better and faster control of the infection, which could be attributed to the presence of pathogen-specific antibodies with higher levels of bacterial killing *in vitro*. Blood clearance resistance has been associated with the thick CPS that protects *S*. *suis* from innate immune system recognition and phagocytosis [14,52,53]. Therefore, antibodies targeting the CPS of encapsulated pathogens can be highly protective by inducing opsonophagocytosis [54,55], a property that has been demonstrated with *S*. *suis* using CPS-specific monoclonal antibodies [16,32]. However, the relative contribution of antibodies targeting either the CPS or other bacterial surface antigens (such as proteins) and the Ig subclasses involved in the control of infection remain poorly understood. Despite having a diversified Th1 and Th2 IgG antibody profile targeting the whole bacteria, our study showed, for the first time, that protective antibodies generated during infection were largely IgM. This observation is supported by the fact that mice having impaired or depleted IgG responses are still able to eliminate *S*. *suis in vivo* and *in vitro*. Unlike IgG, the IgM response shows an apparent increase in avidity in reinfected mice. However, since the IgM antibodies that are relevant for *S suis* opsonophagocytosis do not require AID or GC, we must conclude that either this affinity increase is dispensable for opsonophagocytic activity, or that the increase reflects the expansion of specific clonotypes of higher affinity, rather than the canonical affinity maturation process by SHM.

We found that mice mount weak antibody responses to the CPS of *S*. *suis*, consistent with CPS being poorly immunogenic [31,56]. Furthermore, no IgG and only relatively low titers of IgM targeting the CPS are produced following infection. Similarly, a study reported that the CPS-specific antibody response was low and characterized by the lack of isotype switching after both, primary and secondary infections in pigs [31]. Polysaccharides have long been viewed as TI antigens since they cannot recruit cognate T-cell help, a consequence from not being able to bind to MHC-II and thus be presented to the TCR [57]. However, not all polysaccharides are TI antigens, such as the CPS from *S*. *pneumoniae* serotype 1, that possesses zwitterionic charges that allow binding to MHC-II and cognate T cell activation [58]. This property has not been reported for the CPSs of *S*. *suis*; nonetheless, production of anti-CPS IgMs was substantially reduced but not eliminated by the loss of T cell function in *Tcrb*^-/-^ mice. A possible explanation for this process would be that B cells benefit from the help of non-cognate T cells to build the anti-CPS antibody response. This bystander interaction between T and B cells has been shown to be important for the anti-polysaccharide antibody response in *S*. *pneumoniae* [59,60]. IgM levels targeting the CPS of *S*. *suis* were less affected in SAP mice, indicating that this response has a large extrafollicular component. Finally, antibody-induced killing of *S*. *suis* was significantly affected in *Tcrb* ^-/-^ mice, suggesting a relationship between anti-CPS IgM levels and optimal bacterial clearance.

Overall, our work highlights the importance of IgM antibodies in the elimination of *S*. *suis*. The protective IgM response persisted over day 35 in our model. Therefore, it could reasonable be hypothesized that IgM is important throughout the eradication of *S*. *suis*. This observation rationalizes previous studies describing that *S*. *suis* possesses a protease, Ide_Ssuis_ (Immunoglobulin M-degrading enzyme of *S*. *suis*), that can specifically neutralize swine IgMs [61]. Having such a protease would provide a significant advantage against the immune system by reducing the host ability to counter the bacteria, especially upon reinfection. The same enzyme was also found to be able to neutralize porcine mIgM B cell receptor, which might also further hamper B cell activation and weaken host defences [62]. In agreement with our results using the mouse model, two studies reported that survival of *S*. *suis* in porcine blood was very much restricted in the presence of IgM [63,64]. Indeed, addition of Ide_Ssuis_ protease, cleaving porcine IgM but not IgG, to the bactericidal assays with swine blood, resulted in a pronounced increase in blood *S*. *suis* survival [64]. We must not completely rule out the potential role that IgGs could play against *S*. *suis*. Indeed, *S*. *suis* also possesses a protease able to degrade swine IgGs [65], which indicates that IgGs also exert an evolutionary pressure on *S*. *suis*. Nonetheless, our data point towards approaches that elicit anti-CPS and other anti-*S*. *suis* IgM when envisioning vaccine strategies.

In summary, this study provides an in-depth analysis of the development of the antibody response following *S*. *suis* infections, which help improving future vaccine design. *S*. *suis* impairs optimal maturation of the IgG response, probably limiting their protective capacity, as evidenced herein. It is suggested that IgMs targeting the bacterium and its CPS play a major role during *S*. *suis* infections, an information that could be useful for the development of future effective vaccines.

## Material and methods

### Ethics statement

All mouse procedures were carried out in accordance with the recommendations of the guidelines and policies of the Canadian Council on Animal Care and the principles set forth in the Guide for the Care and Use of Laboratory Animals. Protocols and procedures, including euthanasia to minimize the suffering of animals seriously affected by infection according to an established clinical endpoint grid, were approved by the Animal Welfare Committee of the University of Montreal (Research protocol numbers: Rech-1399 and Rech-1523). Some mouse strains were bred at the Institut de Recherches Cliniques de Montréal (IRCM), as approved by the IRCM Animal Protection Committee protocol 2019–05, before transferring to Faculty of Veterinary Medicine of the University of Montreal. Mice had free access to water and food pellets as well as enrichment such as diverse materials to use as bedding alongside access to a refuge and a rubber toy for gnawing.

### *S*. *suis* strains and growth conditions

The well characterized and highly virulent *S*. *suis* serotype 2 P1/7 strain, originally isolated from a pig with meningitis [66], was used in this study. The strain was grown on sheep blood agar (Oxoid, Fisher Scientific, Ottawa, ON, Canada) and individual colonies were cultured in 5 ml of Todd-Hewitt broth (THB, Becton Dickinson, Mississauga, ON, Canada) for 8 h at 37°C with 120 rpm agitation. The bacterial suspension was then diluted 10^3^ and 10 μl of this dilution were used to inoculate 30 ml of fresh THB, which was cultured for 16 h at 37°C with agitation. Bacteria were then washed twice with phosphate-buffered saline (PBS) pH 7.4 and re-suspended in THB for *in vivo* experimental infections. For *in vitro* infections, washed bacteria were re-suspended in complete cell culture medium, which consisted of RPMI-1640 medium supplemented with 5% heat inactivated fetal bovine serum, 10 mM HEPES, 2 mM L-glutamine and 50 μM 2-mercaptoethanol (Gibco, Burlington, ON, Canada). Bacterial cultures were diluted and plated on THB agar to determine the concentration of colony forming units (CFU) of the final suspensions.

### Mouse experimental infections and immunisations

WT mice (C57BL/6) were purchased from Charles River (Wilmington, MA, USA). In addition, *Aicda*^−/−^ (B6.Cg-*Aicda*^*tm1Hon*^) mice [23], *Ung*^−/−^ (B6;129P2-*Ung*^*tm1Tld*^) mice [35], SAP^−^ (B6.129-*Sh2d1a*^*tm1Lyin*^) mice [67,68] and μMT (B6.129S2-*Ighm*^*tm1Cgn*^) mice [38] in the C57BL/6J background alongside WT counterparts were bred at the specific pathogen-free facility of the Institut de Recherches Cliniques de Montreal (IRCM; Montreal, QC, Canada) and transferred to the Faculty of Veterinary Medicine of the University of Montreal for the experimental infections. *Tcrb*^*−/−*^ (B6.129P2-*Tcrb*^*tm1Mom*^) alongside WT counterparts (C57BL/6J) were purchased from Jackson Research Laboratories (Bar Harbor, ME, USA). All mice were housed under specific pathogen-free conditions.

A well-standardized C57BL/6 mouse model of infection was applied [42,69] using five-week-old mice as *S*. *suis* causes disease in young animals [6]. Mice were acclimatized to laboratory conditions during a whole week before experiments were carried out. Mice were experimentally infected with 1 ml of a 1 x 10^6^ CFU/ml suspension of live *S*. *suis* serotype 2 P1/7 strain administrated by intraperitoneal injection on day 0 with subsequent infections carried out on days 14 and 28, if required for the experiment. Optimal bacterial dose was determined based on previous publications [30,70] to obtain a reliable infection (bacteremia) and limited mortality. For each experiment, negative-control mice were included, where mice were injected with the matching vehicle solution. Blood samples were taken at various time points from either the tail vein or the submandibular vein. Mice were euthanized at different time points, and the serum was collected from the total blood and kept at—80°C for further analysis.

Groups of mice immunized with a formulation composed of 10 μg OVA (Sigma-Aldrich, St. Louis, MO, USA) diluted in PBS and suspended in alhydrogel 2% (Brenntag; Frederikssund, Denmark) were used as positive controls for selected experiments. OVA is a widely used and characterized model antigen commonly employed to study immune responses and it was used in this study as a positive control [15,50,71]. Numbers of mice used in the different experiments are detailed in figure legends.

### Measurement of bacteremia

Blood bacterial loads were assessed in infected mice by collecting 5 μl blood samples from the tail vein at 6 h, 24 h and 48 h post-infection. Samples were serially diluted in PBS and plated on THB agar to count bacterial colonies. If the colony count of the first dilution of a sample was negative, the sample was attributed the minimum value of 100 which corresponds to the lowest dilution factor employed (represented as 10^2^ in the Y axis of figures).

### Titration of *Streptococcus suis* specific antibodies

Enzyme-linked immunosorbent assay (ELISA) was performed as previously described [30]. Microwell plates, Nunc Immuno Polysorp (Thermo Fisher; Rochester, NY, USA), were coated with 100 μl of a 1 x 10^8^ CFU/ml suspension of *S*. *suis* serotype 2 P1/7 strain. Coated plates were air dried, after which 50 μl of 32 M methanol (Sigma-Aldrich) was added and left to evaporate. Dried coated plates were then stored at room temperature (RT) until use. Plates were washed with PBS supplemented with 0.05% Tween 20 (Sigma-Aldrich) (PBS-T). Mouse sera were serially diluted (2-fold) in PBS-T, with 100 μl being added to wells and incubated 1 h at RT. Following PBS-T washes, plates were incubated with either peroxidase-conjugated goat anti-mouse total Ig [IgG + IgM], IgG (Jackson ImmunoResearch; West Grove, PA, USA), IgM, IgG1, IgG2b, IgG2c, or IgG3 (Southern Biotech; Birmingham, AL, USA) antibodies for 1 h at RT. After PBS-T washes, plates were developed with 100 μl of 3,3′,5,5′-tetramethylbenzidine substrate (TMB; Invitrogen; Frederick, MD, USA), and the reaction was stopped by adding 50 μl of 0.5 M H_2_SO_4_ (Fisher Scientific). The reaction was stopped when an OD_450_ value of 1.0 was obtained for the positive internal control comprised of a pool of sera from mice hyperimmunized with *S*. *suis* serotype 2 as previously described [56]. The reciprocal value of the last serum dilution resulting in an absorbance of 0.15 (pre-established cut-off value) was considered the titer of that serum. If the absorbance value of the first dilution of a sample was lower than the cut-off value, the titer was attributed the minimum value of 100 which corresponds to the lowest dilution factor employed (represented as 10^2^ in the Y axis of figures). All antibodies used are listed in S1 **Table**.

### Titration of *Streptococcus suis* capsular polysaccharide-specific antibodies

ELISA was performed as previously described [30] where Nunc Immuno Polysorp microwell plates (Thermo Fisher) were coated with 200 ng of native *S*. *suis* serotype 2 CPS in 0.1 M NaCO_3_ (pH 9.6) (Fisher Scientific) and incubated overnight at 4°C as described previously [30]. Plates were then washed with PBS-T and blocked with PBS containing 1% bovine serum albumin (GE Healthcare Life Sciences, Mississauga, ON, Canada) for 1 h. Mouse sera samples were serially diluted (2-fold) in PBS-T, with 100 μl being added to wells and incubated 1 h at RT. Monoclonal antibodies (mAb) against the *S*. *suis* serotype 2 CPS, mAb 16H11 (IgG) and mAb 9E7 (IgM) were used as positive controls [16]. Following PBS-T washes, plates were incubated with peroxidase-conjugated goat anti-mouse, IgG (Jackson ImmunoResearch) or IgM (Southern Biotech) antibodies for 1 h at RT. The reaction was developed as described above. All antibodies used are listed in **S1 Table**.

### Antibody avidity index assays

The avidity index of mouse sera was determined using the sodium thiocyanate (NaSCN) method described previously [16]. Serum dilutions, in PBS-T, providing readings of at least OD_450_ = 1.0 were incubated in wells coated with either *S*. *suis* serotype 2 P1/7 strain or OVA for 1 h at RT. A gradient (0 to 1 M) of NaSCN (Sigma-Aldrich) was added to the wells for 15 min at RT. The integrity of *S*. *suis* and OVA coatings following NaSCN treatment was tested using control plates. NaSCN treatment did not affect coating integrity. ELISA for anti-OVA antibodies was performed as previously described [30] and used as a control. Following treatment, NaSCN and eluted antibodies were removed by PBS-T washes. Peroxidase-conjugated goat anti-mouse IgG (Jackson ImmunoResearch) were added to wells and incubated for 1 h at RT. After PBS-T washes, plates were developed with TMB, and the reaction was stopped by adding 50 μl of 0.5 M H_2_SO_4_. Absorbance of wells was read at 450 nm with an ELISA plate reader. The avidity index of sera is the concentration of NaSCN at with 50% of *S*. *suis*-specific or OVA-specific antibodies are eluted.

### Depletion of IgGs from mouse sera

To deplete the IgG content of mouse sera, Dynabeads Protein G (Invitrogen) were used on pooled sera from WT mice previously infected 3 times with *S*. *suis* P1/7 strain. Briefly, 5 ml of beads were transferred to a microtube that was then placed on the magnet. The storage solution was removed, and beads were then washed once with sterile PBS, once with EtOH 70% and then washed twice with sterile PBS. The remaining liquid was then removed from the beads and 1 ml of the pooled sera was added. Following 30 minutes of incubation at RT with mild manual agitation, the sera was removed from the beads and transferred to a new microtube. Before further experiments, IgG removal was validated by comparing the treated sera with untreated sera by ELISA titration against whole *S*. *suis* (as described above). Bead treatment successfully removed more than 97% of IgGs and less than 6% of IgMs.

### Antibody-dependent *in vitro* opsonophagocytosis assay (OPA)

OPA assay was performed as described previously [72]. OPA is based on the fact that opsonization by specific immunoglobulins (antibodies) at the bacterial surface will be recognized by Fc receptors, triggering an enhanced immune response by blood leukocytes which results in bacterial phagocytosis and bactericidal activity. Instead of using a cell line or a purified single cell type, the OPA test used herein requires whole blood from naive mice. This model takes into account all blood leukocytes present and thus represents a more realistic model of the complex interactions between all immune cells and bacteria during a systemic infection [72]. Briefly, blood from uninfected C57BL/6 mice was treated with sodium heparin (Sigma-Aldrich) after which it was diluted with RPMI complete medium to reach 6.25 x 10^6^ leukocytes/ml. Bacterial preparations suspended in complete cell culture medium were prepared as described above and diluted to obtain 1.25 x 10^6^ CFU/ml. Blood and bacteria preparations were mixed with 40% (vol/vol) of serum from infected or uninfected mice to reach 5 x 10^5^ leukocytes and 5 x 10^4^ CFU of *S*. *suis* in a final volume of 0.2 ml. Tube tops were pierced using a sterile 25-gauge needle, after which the microtubes were incubated at 37°C with 5% CO_2_ for 4 h with light manual agitation every 20 minutes. Samples were collected at 4 h of incubation to perform viable bacterial counts onto THB agar plates. Naïve rabbit serum or reference rabbit anti-*S*. *suis* serotype 2 serum were used as negative and positive controls, as previously described [72]. The bacterial killing percentage was calculated by using the following formula:

$$\%\;of\;bacteria\;killed=\left[1-\frac{CFU\;of\;tested\;sera}{CFU\;of\;negative\;control\;sera}\right]$$

### Flow cytometry analysis of splenic germinal center B cells

The spleens of mice were collected at various time points post-infection, as detailed in figure legends. Splenic cells were passed through a 70 mm cell strainer (Fisher Scientific), centrifuged, and then treated with Ammonium-Chloride-Potassium (ACK) red blood cell lysing buffer (Gibco). Following washes, 4 x 10^6^ total spleen cells were incubated with anti-mouse CD16/CD32 (Fc Block, BD Biosciences; Mississauga, ON, Canada) for 15 min on ice according to manufacturer’s instructions. Afterwards, surface staining with the following antibodies: anti-CD95^PE^, anti-CD86^biotin^, anti-CD19^PE-Cy7^, anti-GL-7^AF647^ and anti-CD184^BV421^ (all BD Biosciences) was performed for 30 min on ice. Following washing, cells were incubated with Brilliant Stain Buffer (BD Biosciences) and stained with Streptavidine^BV605^ (BD Biosciences) for 10 min on ice. Cells were then washed and immediately analyzed on a BD FACSaria Fusion. The data collected was analyzed on FlowJo version 10. All antibodies used are listed in **S1 Table**.

### Statistical analyses

All statistical analyses were done using SigmaPlot version 11.0. A minimum of *p* < 0.05 was considered as statistically significant. To evaluate statistical differences between two paired groups, parametric data were analyzed using a paired *t*-test and non-parametric data were analyzed using a Signed rank test. For unpaired groups, parametric data were analyzed using unpaired *t*-test and non-parametric data were analyzed using Mann-Whitney rank sum test. Significative differences were indicated with an asterisk (*).

To evaluate statistical differences between three groups or more, parametric data were analyzed using one-way analysis of variance (ANOVA) followed by the Tukey Method. Non-parametric data were analyzed using Kruskal-Wallis One-Way ANOVA followed by Dunn’s Method. Groups not sharing a letter (a, b or c) were significantly different.

## Supporting information

S1 Fig Gating strategies for splenic germinal center B cells. Spleens from C57BL/6 mice were analyzed by flow cytometry. Germinal center (GC) B cells were identified as CD19^+^, GL-7^+^ and CD95^+^ with further identification of Dark zone (DZ) GC B cells defined as CD19^+^, Gl7^+^, CD95^+^, CD184^+^ and CD86^int^, and Light zone (LZ) GC B cells defined as CD19^+^, Gl7^+^, CD95^+^, CD184^-^ and CD86^+^.
(TIF)

S2 Fig Splenic B cell populations post immunisation. C57BL/6 mice were infected once (1x) with a 1 x 10^6^ CFU dose of live *S*. *suis* serotype 2 strain P1/7. (A) Percentages of B cells in the spleen and germinal center (GC) B cells in the B cell population (B) evaluated by flow cytometry at various timepoints in the spleens of uninfected (*n*; D0 = 8, D7 = 6, D10 = 6, D13 = 3, D21 = 6, D28 = 3, D35 = 4, D44 = 4, D70 = 3 and D98 = 5), *S*. *suis*-infected (*n*; D0 = 8, D7 = 5, D10 = 5, D13 = 18, D21 = 16, D28 = 5, D35 = 5, D44 = 5, D70 = 5 and D98 = 4) and ovalbumin-immunized (*n*; D0 = 8, D7 = 8, D10 = 10, D13 = 3, D21 = 7, D28 = 5, D35 = 3, D44 = 3, D70 = 7 and D98 = 3) mice. Data are represented as individual plots and median.
(TIF)

S3 Fig Avidity of IgG classes following *S*. *suis* infection. C57BL/6 mice were infected with a 1 x 10^6^ CFU dose of live *S*. *suis* serotype 2 strain P1/7. Avidity index of *S*. *suis*-specific IgGs in the sera of mice infected once (*n* = 5), twice (*n* = 5) or thrice (*n* = 6) with sera collection on day 35 for all groups and then evaluated by ELISA coupled with the use of the chaotropic agent NaSCN. The avidity index represents the millimolar concentration of NaSCN at which 50% of antigen-specific antibodies are eluted. Data are represented as individual values with mean. No significant difference was observed between groups by Student’s *t*-test.
(TIF)

S1 Table Antibodies used. (XLSX)

S1 Appendix Supporting data. (XLSX)
